# Non-linearity correction in NIR absorption spectra by grouping modeling according to the content of analyte

**DOI:** 10.1038/s41598-018-26802-w

**Published:** 2018-06-04

**Authors:** Ai Liu, Gang Li, Zhigang Fu, Yang Guan, Ling Lin

**Affiliations:** 1 State Key Laboratory of Precision Measurement Technology and Instruments, Tianjin University, Tianjin, 300072 China; 2 Tianjin Key Laboratory Biomedical Detecting Techniques and Instruments, Tianjin University, Tianjin, 300072 China; 3 Med Examinat Ctr, 254 Hosp Peoples Liberat Army, Tianjin, 300142 China

## Abstract

To correct the non-linearity caused by light scattering in quantitative analysis with near infrared absorption spectra, a new modeling analysis method was proposed: grouping modeling according to the content of analyte. In this study, we tested the proposed method for non-invasive detection of human hemoglobin (Hb) based on dynamic spectrum (DS). We compared the prediction performance of the proposed method with non-grouping modeling method. Experimental results showed that the root mean square error of the prediction set (RMSEP) by the proposed method was reduced by 9.96% and relative standard deviation of the prediction set (RSDP) was reduced by 4.73%. The results demonstrated that the proposed method could reduce the effects of non-linearity on the composition analysis by spectroscopy. This research provides a new method for correcting the non-linearity stemming from light scattering. And the proposed method will accelerate the pace of non-invasive detection of blood components into clinical application.

## Introduction

The quantitative analysis with absorption spectra has been widely used in medicine^1,2^, chemical^3,4^, food^5,6^, agriculture^7,8^ and other fields, owing to its rapidity, non-destructivity and low cost.

Lambert-Beer’s law is the theoretical basis of quantitative analysis with absorption spectra and it makes the assumption that the absorbing medium doesn’t scatter light^9^. In reality, however, the measured subjects have scattering or even strong scattering properties, for example, milk^10^ and biological tissue^11–14^ have strong scattering properties. Light scattering is a significant factor leading to the non-linear relationship between the absorption spectra and the content of analyte^9,15^, which would seriously decrease measurement accuracy^16^. Therefore, it limits the further improvement and application of quantitative analysis based on NIR absorption spectra. Many researchers are devoted to the study concerning scattering properties of measured subjects^12,14,17–19^ and attempt to correct the non-linearity caused by scattering. Geladi proposed multiplicative scatter correction (MSC) to preprocess the spectra^16^. Other methods including extended MSC (EMSC)^20^, piece-wise MSC (PMSC)^21^, inverted signal correction (ISC)^22^, extended ISC (EISC)^23^ and so on were derived from MSC subsequently. Their common drawback may be that any changes to sample set needs recalibration. Barnes proposed standard normal variate (SNV) transformation^24^ and proved the linear correlativity between SNV and MSC later on^25,26^. Furthermore, comparing with MSC, SNV may be superior because it’s applied to individual spectrum and without any reference to the total sample set^26^, though two methods usually gave almost the same results^22,27^. Miller and Naes proposed a path length correction method (PLC-MC) and demonstrated that better predictions of analyte content were obtained with spectra corrected with PLCMC than with MSC when spectral variability from chemical variations was very large^28^. Wold proposed orthogonal signal correction (OSC)^29^, based on which direct orthogonalization (DO)^30^, orthogonal projections to latent squares (O-PLS)^31^ and direct orthogonal signal correction (DOSC)^32^ and so on were developed; but O. Svensson compared six methods concerning OSC and concluded that these methods didn’t lead to significantly improved prediction accuracy and their advantages just lied in enhancing interpretability of data and reducing the number of principal components to simplify the model^33^. Leger proposed path length distribution correction (PDC) based on time-of-flight (TOF) distribution and the method gives improved RMSEP by 27%, but it does have an important limitation: a path length distribution has to be assumed for each sample, whether it is measured or estimated^9^. There also have been researchers who use non-linear modeling methods including stepwise polynomial PCR (SWP-PCR), stepwise polynomial PLSR (SWP-PLSR) and artificial neural networks (ANN) and other methods to correct the non-linearity stemming from light scattering^34–36^. However, overfitting is easy to occur with respect to the number of principal components^37^ when using SWP-PCR. As for ANN, it suffers from three main drawbacks^35,37^: (1) the predictive properties of ANNs strongly depend on the learning parameters and the topology of the network; (2) the modeling process of ANN tends to be computationally intensive and time-consuming; (3) ANNs models are complex and difficult to interpret. So far, any ideal methods haven’t come out yet, for correcting the non-linearity in non-invasive detection of human blood components with NIR absorption spectra.

It is much more difficult to detect human blood components non-invasively than other analytes, because signal-to-noise ratio (SNR) of detecting human blood components is significantly lower^38^. Although “dynamic spectrum” theory could reduce the influence of individual differences and changes of measurement conditions on the measurement^39^ and has made great progress in signal acquisition and processing^40,41^, dynamic spectrum extraction^42–44^ and modeling^45,46^, non-linear problem caused by scattering still exists. It severely slows the process of clinical application of DS. To correct non-linearity, a new method is proposed in this paper: grouping modeling according to the hemoglobin content. This method can improve the non-invasive measurement accuracy of blood components based on DS.

## Theory

### Dynamic spectrum

**Figure 1:**
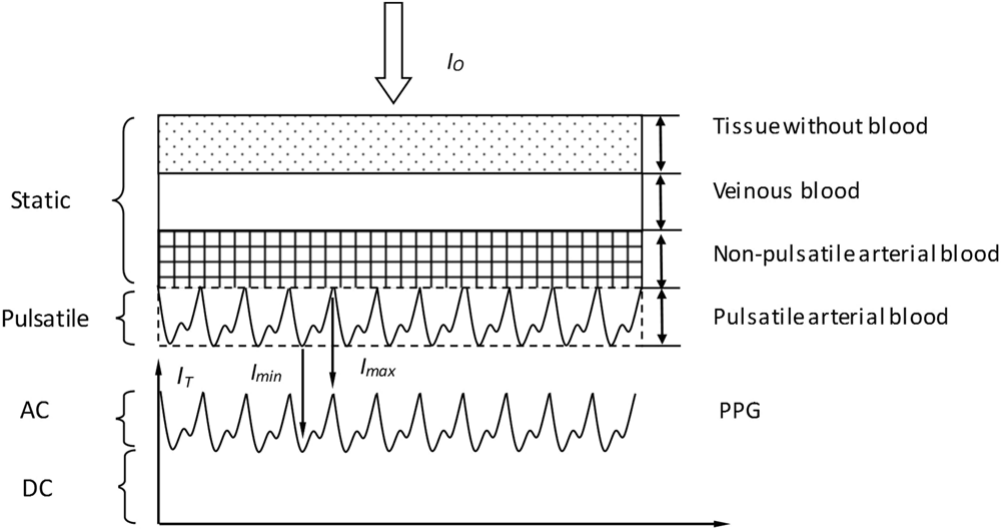
The principal of dynamic spectrum. A simplified model of tissue is shown and the tissue can be regarded as a combination of a pulsatile part and a static part. So, the PPG waveform consists of two parts: AC (pulsatile) and DC (baseline)^49,54,55^. *I*_*T*_ represents transmitted light intensity and *t* represents time.

Dynamic spectrum(DS)^39^ is a theory and method for the non-invasive measurement of human blood components based on photoplethysmography (PPG)^47,48^. The essence of DS is to derive the difference between the maximum and minimum absorbance, within one single period of PPG and at each single wavelength. Its advantage lies in that individual differences caused by skin, muscle and so on are eliminated in a certain degree, by calculating the absorbance difference between arterial systole and diastole^39,49^. The principal of DS is shown as Fig. 1.

Supposing there is an incident light *I*_*o*_^44,45^. When the artery filling reaches a minimum state, the incident light is not subjected to pulsatile arterial blood. At this time, the output light intensity will be the strongest (referred as *I*_*max*_), which can be regarded as the incident light *I*_o_ of pulsatile arterial blood. When the artery filling reaches the highest state, effects of pulsatile arterial blood have reached the strongest. At this time, the output intensity will be the weakest (referred as *I*_min_) and it can be regarded as the minimum output intensity of pulsatile arterial blood. Therefore, by recording the absorbance value of both the maximum value in arterial systole and the minimum value in arterial diastole, the effect of skin and subcutaneous tissue can be eliminated, whose absorption can be supposed to be constant. According to modified Lambert-Beer’s law, the formula of absorbance and absorbance difference is as equations (1) and (2). So, the ∆*OD* at all wavelengths (∆*OD*^*λ1*^, ∆*OD*^*λ2*^, ∆*OD*^*λ3*^, …, ∆*OD*^*λn*^) can be regarded as the spectrum of the pulsatile arterial blood and it is named as Dynamic Spectrum (DS for short).

1 $$O{D}^{\lambda }=\mathrm{log}({{I}_{i}}^{\lambda }/{{I}_{0}}^{\lambda })=-{\sum _{i}{\varepsilon }_{i}}^{\lambda }{c}_{i}{B}^{\lambda }l+G$$

2 $${\rm{\Delta }}O{D}^{\lambda }=O{D}_{\max }^{\lambda }-O{D}_{\min }^{\lambda }=\mathrm{log}({I}_{\max }^{\lambda }/{I}_{\min }^{\lambda })$$

*OD* is the absorbance difference in a cardiac cycle, *ε*_*i*_^*λ*^ is the molar extinction coefficient of the ith wavelength, *c*_*i*_ is the content, *l* is the optical path length and *G* is the scattering loss.

### Theoretical basis of grouping modeling according to the content of analyte

Quantitative analysis with absorption spectra is based on a very important premise that Lambert-Beer’s law can be applied. In other words, there exists a linear relationship between absorption spectra and the content of analyte. But in fact, though light scattering spoiled this linearity, there still exists a monotone non-linear relationship between absorption spectra and the content of analyte. In this paper, Partial Least Squares Regression (PLSR) was used as the modeling method, which is one of the most popular methods in NIR multivariate calibration^50^. It works with the whole spectrum, by synthesizing it into a series of linearly-independent variables^36^. The calculation of these variables is based not only on spectral data but also on reference values for the parameter measured in each sample. A most valuable feature of PLSR is that it deals very well with the problem of collinearity with overdetermined linear systems^50^. Its another distinct advantage is that it obviates the need to select wavelengths for model development^36^.

**Figure 2:**
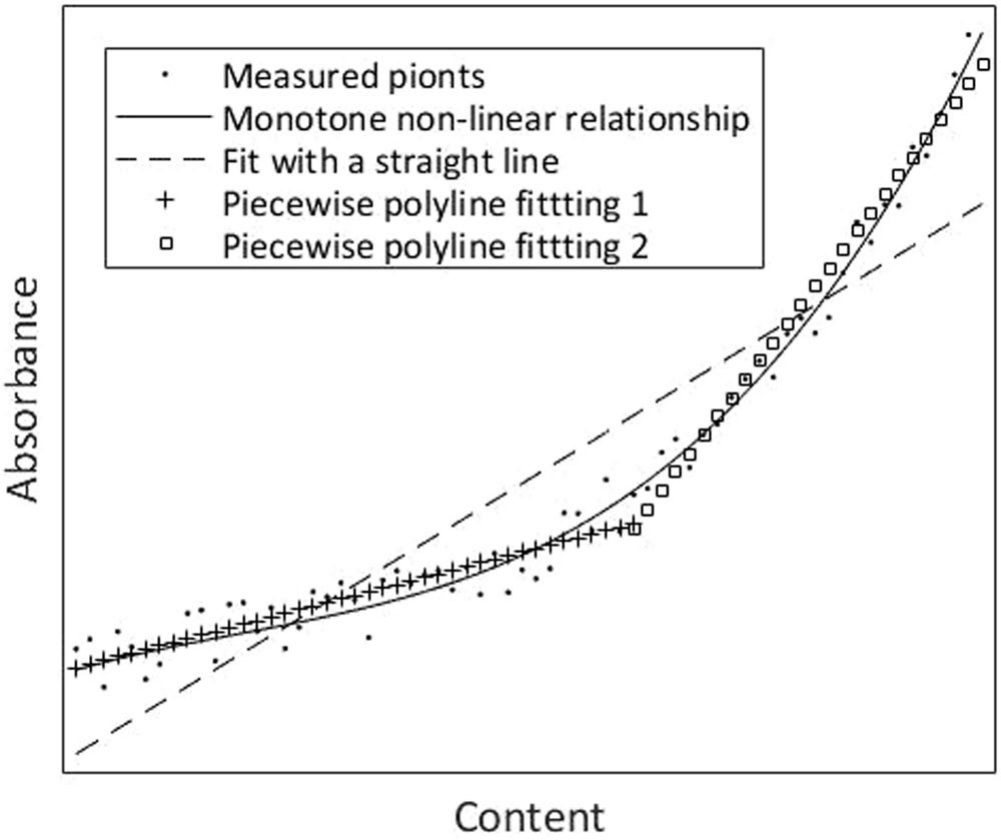
Piecewise polyline fitting of a curve in multi-dimensional space (also schematic of grouping modeling).

As shown in Fig. 2, the absorbance at each wavelength of interest and the content of analyte constitute a multi-dimensional space. The above-mentioned monotone non-linearity can be expressed with a curve line in this space, roughly as the solid line in Fig. 2. Because of measurement errors, the actual absorbance and content are shown as scatter points in Fig. 2. PLSR is essentially equivalent to using a straight line (as the dotted line in Fig. 2) to fit these measuring points in the multi-dimensional space (or more actually, using a straight line to fit principal components synthesized with these measurement points). This method will undoubtedly lead to great errors owing to the existence of non-linearity. But if we divide samples into two or more groups, in other words, two or more straight lines (as lines marked with “+” and “γ” in Fig. 2) are used to perform piecewise polyline fitting of a curve, the accuracy must be higher than that with a single straight line. Here, we proposed “grouping modeling” to correct the non-linearity between absorption spectra and the content of analyte. The above-mentioned content also explains why grouping modeling according to the content of analyte can improve measurement accuracy.

In the qualitative analysis based on absorption spectra, absorption spectra are the input variables and contents of analyte are the output variables. So, we are more inclined to the assumption that if grouping is based on absorption spectra, we will know which grouping model should be used to predict the content, after getting a new spectrum from one sample. But as we all know, the absorption spectrum is a multi-dimensional vector (often dozens or even hundreds of dimensions), which make it not so easy to group based on spectra. Consequently, we choose grouping based on contents of analyte. However, there still exists a problem: for an unknown sample, we don’t know the content of analyte to be predicted, so we can’t determine which grouping model should be used. Here, we find a relatively reasonable solution: After establishing grouping models, we establish a non-grouping model to get a preliminary prediction of the content. By doing this, we can determine which grouping model should be used for each sample to get a second prediction. The detailed steps of grouping modeling are described in the following section “Non-grouping modeling and grouping modeling”.

### Non-grouping modeling and grouping modeling

#### Non-grouping modeling

When modeling, most researchers don’t divide samples into groups. To be distinguished from the new proposed method “grouping modeling”, here we give a name “non-grouping modeling” to the commonly used method, also for convenience of description.

**Figure 3:**
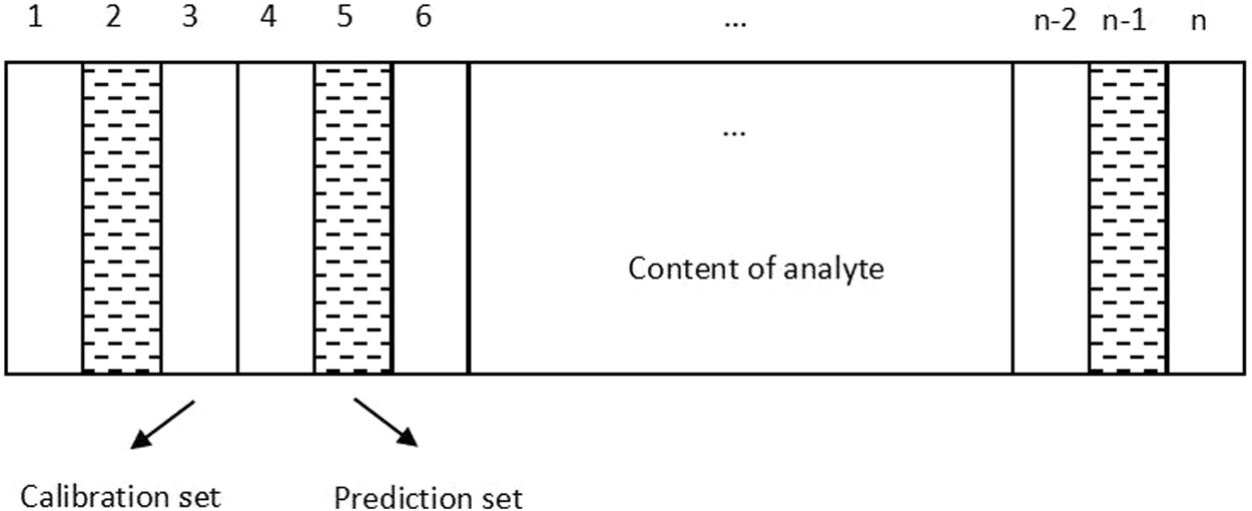
Schematic of the calibration set selection in non-grouping modeling. Note: n represents the number of all samples.

There is just one calibration set (named as Total calibration set) and one prediction set (named as Total prediction set) in non-grouping modeling. The steps of non-grouping modeling are listed as follows. Firstly, sort all samples according to the content of analyte. Secondly, select the calibration set and prediction set with ensuring the content range of analyte in the calibration set covers that in the prediction set^46,51^, roughly as shown in Fig. 3. Finally, establish the calibration model.

#### Grouping modeling

The detailed steps of grouping modeling are listed as follows:

1. Sort all samples according to the content of analyte and select suitable number of samples as the prediction set (Total prediction set). The remaining is the calibration set (Total calibration set) to be grouped. It should be ensured that the content range of analyte in the calibration set covers that in the prediction set.
2. Divide the Total calibration set into a certain number of groups in ascending order of the content of analyte. The range of content varies between different groups. Then, establish the grouping model based on the calibration set of each group separately, named as grouping model 1, grouping model 2, and so on. The number of groups to be divided depends on the number of samples and the range of analyte content.
3. Establish a non-grouping model with the Total calibration set. Put samples of Total prediction set into this model to get preliminary predictions and then determine which grouping model should be used for these samples.
4. Put the Total prediction set into corresponding grouping models to get a second prediction and compare the prediction results between grouping modeling and non-grouping modeling.

## Method

### Experimental device

**Figure 4:**
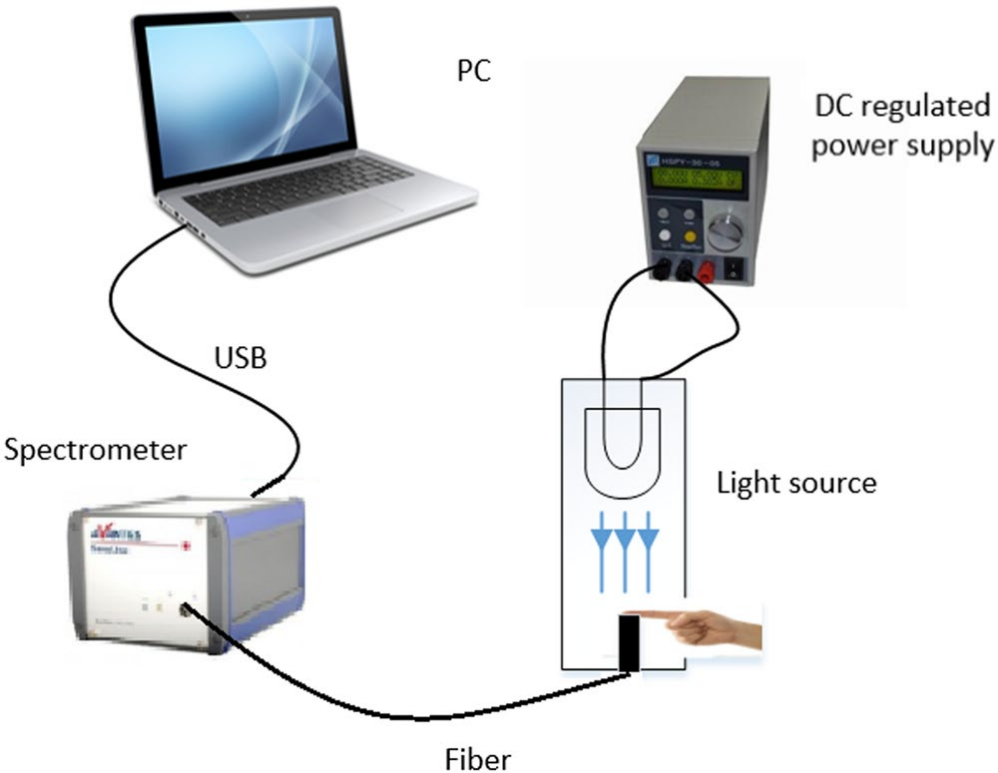
Experimental device.

The experimental device is composed of a bromine tungsten lamp, a programmable voltage regulator, a near-infrared spectrometer, an optical fiber and a portable computer, as shown in Fig. 4. The programmable regulator HSPY-30-05 supplies power to the bromine tungsten lamp with DC voltage of 12 V; bromine tungsten lamp scatters light through the fingertip which then received by AvaSpec-HS1024x58TEC spectrometer with the wavelength range of 200–1160 nm. 591–1044 nm is used in this work and the spectrometer transmits data to the computer via USB.

### Samples and measurements

The experiments were carried out in 254 Hosp Peoples Liberat Army. Subjects of the experiments were recruited from the people who were going to accept a blood routine examination in the hospital.

**Figure 5:**
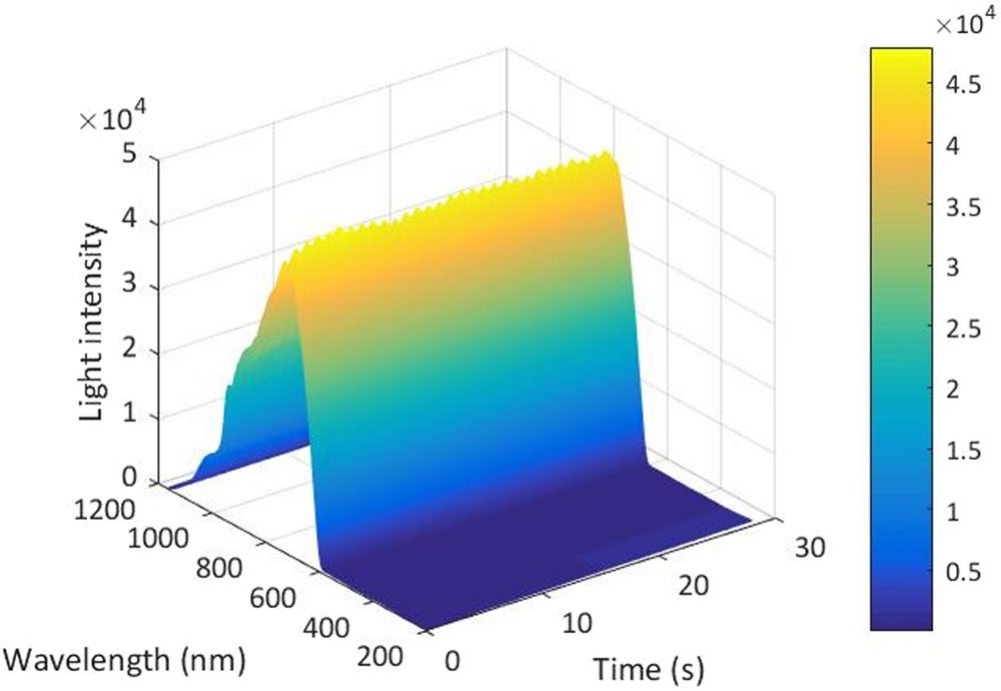
Original signals sampled by the spectrometer of one subject.

During the experiment, fingertip of each subject completely covered the entrance of optical fiber, with contact pressure remaining stable. The integration time of spectrometer was 20 ms and the measurement lasted for 30 s. After the experiment, subjects took blood routine examination to obtain Hb contents. The blood samples were tested with a fully automated hematology analyzer (ABX Pentra 60, manufactured by HORIBA ABX SAS, Japan) in the hospital. Then sampled data by the spectrometer were made to format conversion via Avaspec software (version 76USB2). After eliminating the abnormal samples, 275 samples were used to establish models. All calculations were achieved in MATLAB (version R2016a). Original data of one sample from the spectrometer is shown as Fig. 5.

### Ethics statement

All the subjects gave their informed consent to the study participation. All these experiments were conducted in compliance with the relevant laws as well as the guidelines issued by the Ethical Committee of Tianjin University and 254 Hosp Peoples Liberat Army. The experiments also got the approval of the Ethical Committee of Tianjin University and 254 Hosp Peoples Liberat Army.

### DS extraction method

**Figure 6:**
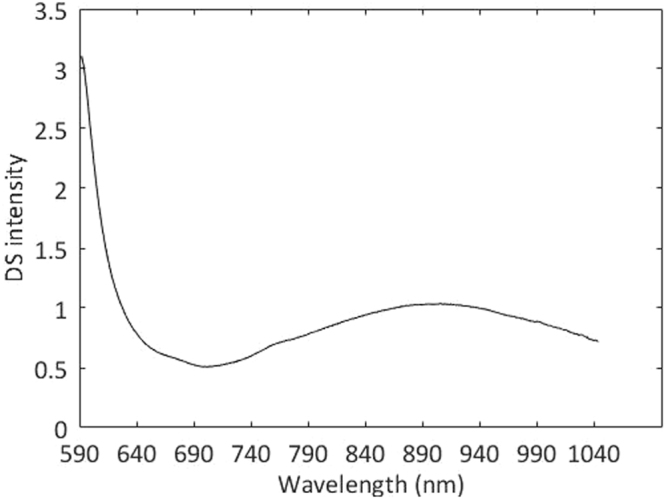
DS signal extracted from one sample.

Dynamic spectrum was extracted by single-trial estimation^52^, which performs well comprehensively in noise suppression and extraction accuracy of DS. The DS signal extracted from one sample is shown in Fig. 6. Then we established the calibration model between DS and Hb content with PLSR.

### Data Availability

The datasets generated during and/or analyzed during the current study are available from the corresponding author on reasonable request.

## Results and Discussions

The process of grouping modeling is as follows

1. All 275 samples were sorted according to the content of Hb, 48 samples were taken out to constitute the Total prediction set and the remaining 227 samples worked as the Total calibration set. The range of Hb content in the calibration set covered that in the prediction set.
2. Samples in the Total calibration set were sorted by Hb content and divided into two groups. The range of Hb content is 107–176 g/L in the Total calibration set and it was divided into three parts evenly. Samples in the first two parts were taken as the calibration set of group 1, with the Hb content at the range of 107–154 g/L. Samples in the last two parts were taken as calibration set of group 2, with the Hb content at the range of 130–176 g/L. The number of samples and the range of Hb content are listed in Table 1. The second part is the overlapping section between the calibration set of group 1 and the calibration set of group 2, which is applied to ensure that each group has enough samples, since limited samples may affect the robustness of models. Then, the models were established based on the calibration set of each group separately with PLSR and therefore grouping model 1and grouping model 2 were obtained. The distribution of Hb content of the Total calibration set, the calibration set of group 1 and group 2 are shown in Fig. 7.

**Table 1:** The number of samples and content range in the Total calibration set, Total prediction set, calibration set of group 1 and group 2, prediction set of group 1 and group 2.

	Total calibration set	Calibration set of group 1	Calibration set of group 2	Total prediction set	Prediction set of group 1	Prediction set of group 2
Content range (g/L)	107–176	107–154	130–176	112–169	112–139	121–169
Number of samples	227	182	192	48	9	39

**Figure 7:**
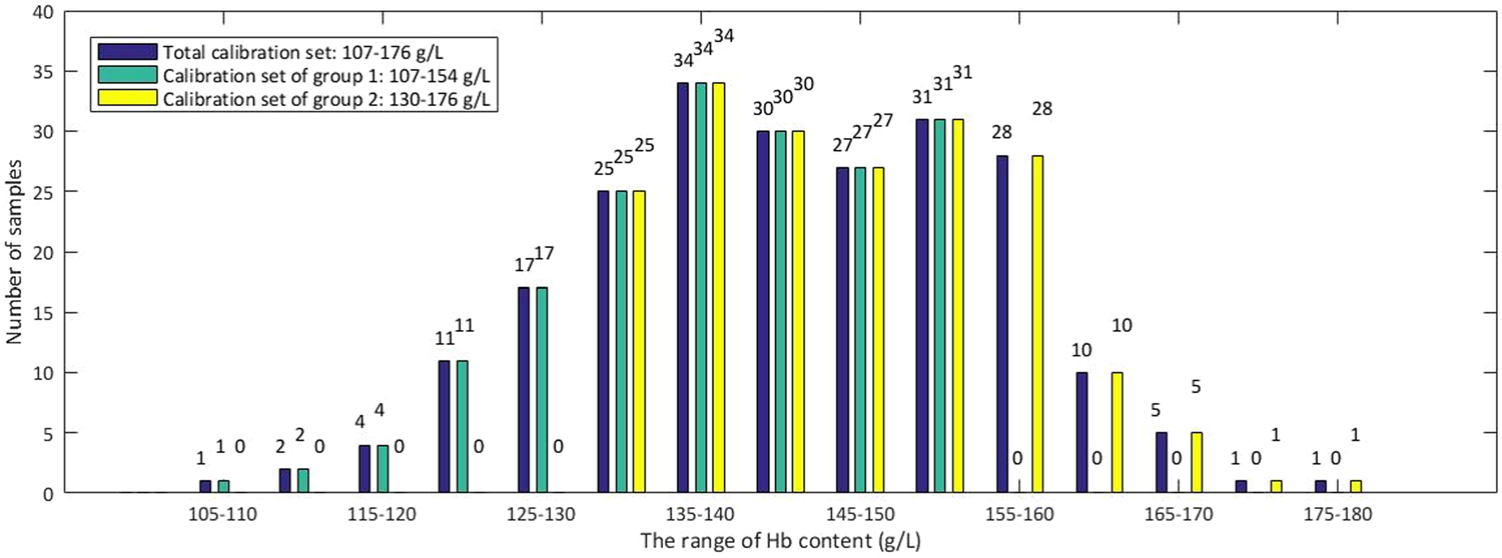
Distribution of Hb content of the Total calibration set, the calibration set of group 1 and group 2.
3. A non-grouping model was established with the Total calibration set by PLSR. Samples of the Total prediction set were put into this model to get preliminary predictions of Hb content and then we could determine which grouping models should be used for these samples. A scatter plot of true value and predicted value of Hb content is shown in Fig. 8.

**Figure 8:**
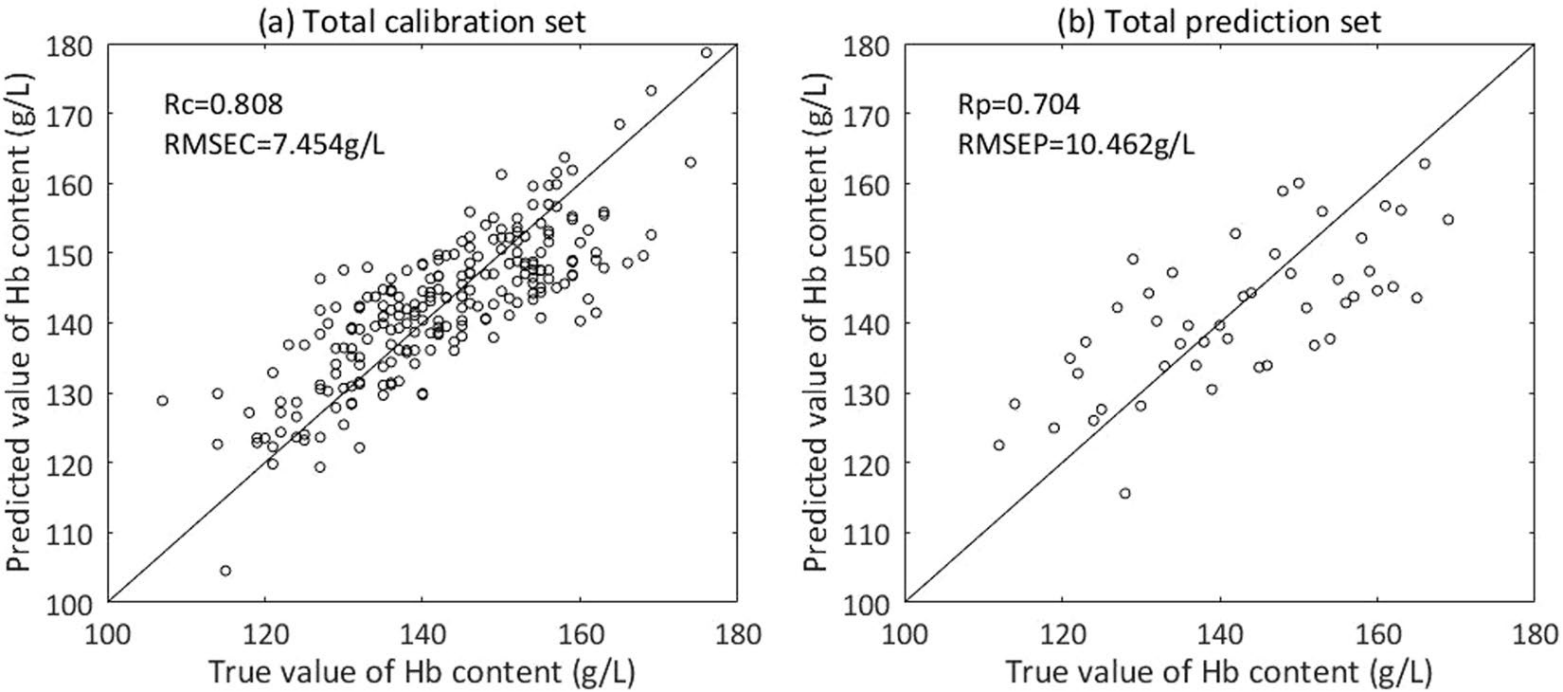
Scatter plot of true value and predicted value of Hb content by non-grouping modeling. (**a**) Total calibration set. (**b**) Total prediction set.
4. Then, samples in the Total prediction set should be predicted for a second time with grouping model 1 or grouping model 2. Firstly, we need to decide which grouping model work better for each sample in the Total prediction set, especially for the samples within the range of 130–154 g/L. Here, we define a certain value of Hb content as the threshold content. When Hb content of samples is lower than the threshold, the samples should be predicted by grouping model 1, when higher, by grouping model 2. To find the threshold content, we calculated the Total RMSEP of two groups with different thresholds of Hb content, from 130 g/L to 154 g/L. The content which makes the Total RMSEP of two groups smallest is chosen as the threshold content, namely 133 g/L. Samples whose prediction results by the non-grouping modeling were lower than 133 g/L were predicted again by grouping model 1, and the remaining by grouping model 2. The distribution of Hb content of the Total prediction set, the prediction set of group 1 and group 2 are shown in Fig. 9. A scatter plot of true value and predicted value of Hb content in group 1 and group 2 is shown in Fig. 10.

**Figure 9:**
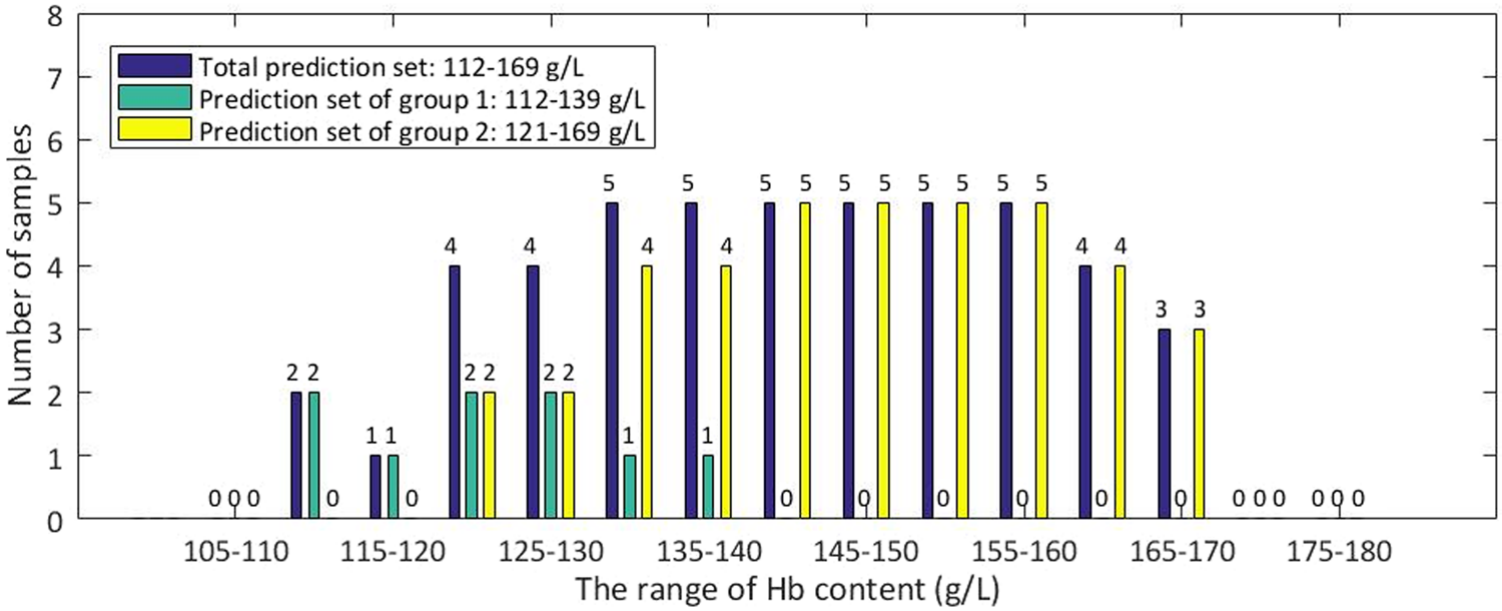
Distribution of Hb content of the Total prediction set, the prediction set of group 1 and group 2.

**Figure 10:**
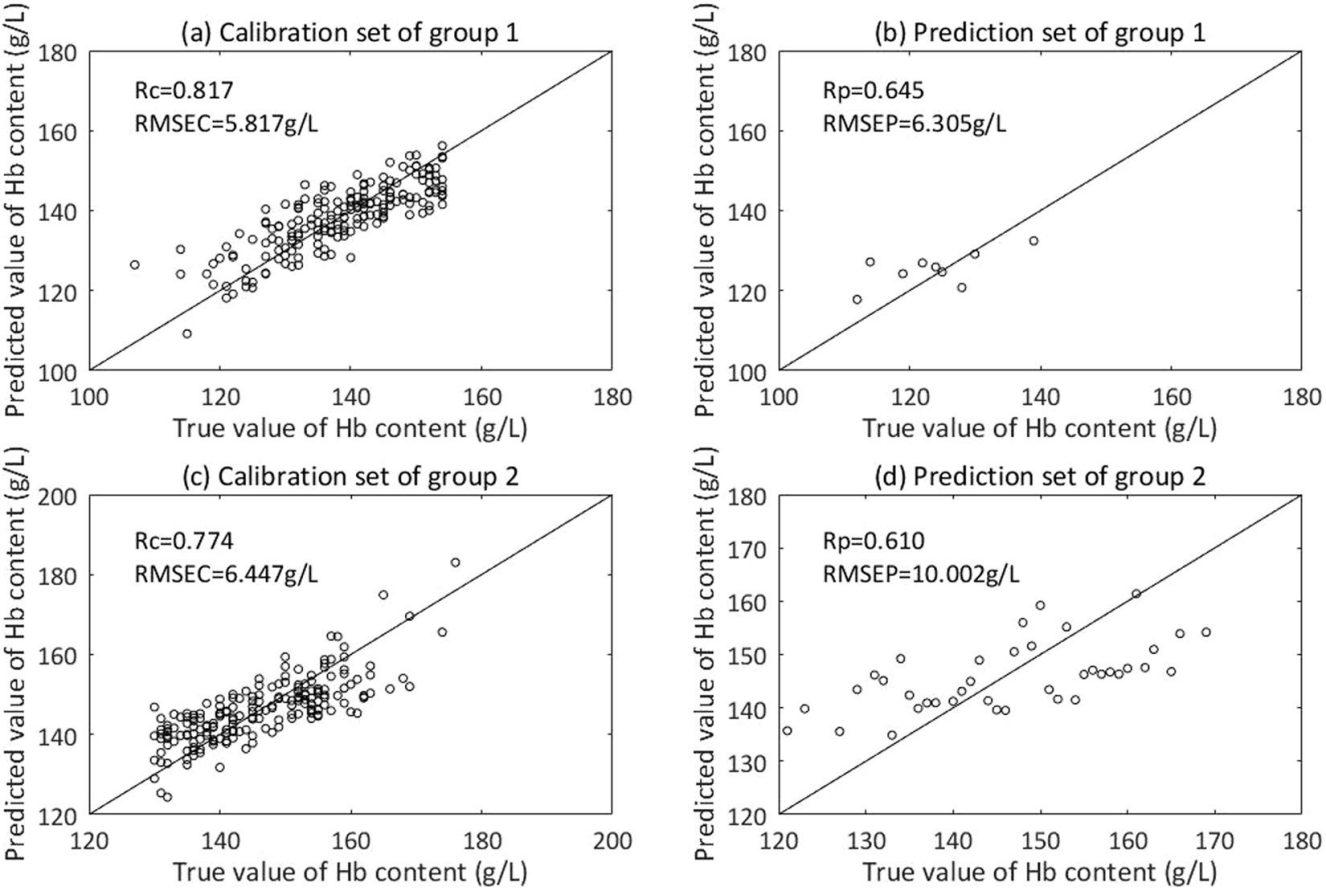
Scatter plot of true value and predicted value of Hb content by grouping modeling. (**a**) Calibration set of group 1. (**b**) Prediction set of group 1. (**c**) Calibration set of group 2. (**d**) Prediction set of group 2.
5. The root mean square error of the calibration set (RMSEC), relative standard deviation of the calibration set (RSDC), root mean square error of the prediction set (RMSEP), relative standard deviation of the prediction set (RSDP) were used as the indexes to evaluate the performances of the developed calibration models, as shown in equations (3) and (4).

3 $$RMSE=\sqrt{\frac{1}{N}\sum _{i=1}^{N}{({y}_{i}-{\hat{y}}_{i})}^{2}}$$

4 $$RSD=\frac{\sqrt{\frac{1}{N}\sum _{i=1}^{N}{({\hat{y}}_{i}-\frac{1}{N}\sum _{i=1}^{N}{\hat{y}}_{i})}^{2}}}{\frac{1}{N}\sum _{i=1}^{N}{y}_{i}}\times 100 \% $$

where N denotes the number of samples, ${y}_{i}$ denotes the true value of Hb content and ${\hat{y}}_{i}$ denotes the prediction value of Hb content.

Meanwhile, relative standard error (RSD) of the reference method (a fully automated hematology analyzer–ABX Pentra 60) is smaller than 1%^53^, which also acts as an evaluating indicator. The evaluation results are shown in Table 2.

**Table 2:** Results comparison between non-grouping modeling and grouping modeling.

Method	PC	*Rc*	RMSEC (g/L)	RSDC	*Rp*	RMSEP (g/L)	RSDP
Non-grouping modeling	8	0.808	7.454	7.162%	0.706	10.462	7.287%
Grouping modeling	x	x	6.148	6.283%	x	9.420	6.942%
Group 1	8	0.817	5.817	5.958%	0.645	6.305	3.305%
Group 2	8	0.774	6.447	5.385%	0.610	10.002	4.225%

Note: PC: number of principal components; *Rc*: correlation coefficient of calibration set, *Rp: c*orrelation coefficient of prediction set.

In Table 2, we can see that RMSEC of group 1 and group 2 are both smaller than that of non-grouping modeling. Total RMSEC of two groups (6.148 g/L) is smaller than that of non-grouping modeling (7.454 g/L) by 17.52%, which means that grouping modeling makes the regression between dynamic spectra and Hb contents better in comparison with non-grouping modeling. This result is consistent with the theoretical analysis about why grouping modeling can improve accuracy in the section “Theoretical basis of grouping modeling according to the content of analyte”. Given that relative standard deviation (RSD) can reflect the credibility of measurement better, we compare RSDC between grouping modeling and non-grouping modeling: RSDC of group 1 and group 2 both are smaller than that of non-grouping modeling and total RSDC of two groups (6.283%) is smaller than that of non-grouping modeling (7.162%) by 12.27%. Therefore, it can be concluded that grouping modeling method, namely dividing the calibration set into groups, we can correct the non-linearity between dynamic spectra and Hb contents in a certain degree.

Table 2 also indicates that RMSEP of group 1 and group 2 are smaller than that of non-grouping modeling by 39.73% and 4.40% respectively. Total RMSEP of two groups (9.420 g/L) is smaller than that of non-grouping modeling (10.462 g/L) by 9.96%. As above-mentioned, grouping modeling makes the regression between dynamic spectra and Hb contents better in comparison with non-grouping modeling, which naturally leads to improved prediction accuracy. And the total RSDP of two groups (6.942%) is smaller than that of non-grouping modeling (7.287%) by 4.73% and RSDP of group 1 and group 2 are both smaller than that of the non-grouping method remarkably. We can also see that, compared to non-grouping modeling method, grouping modeling method are closer to the reference method in RSD.

**Figure 11:**
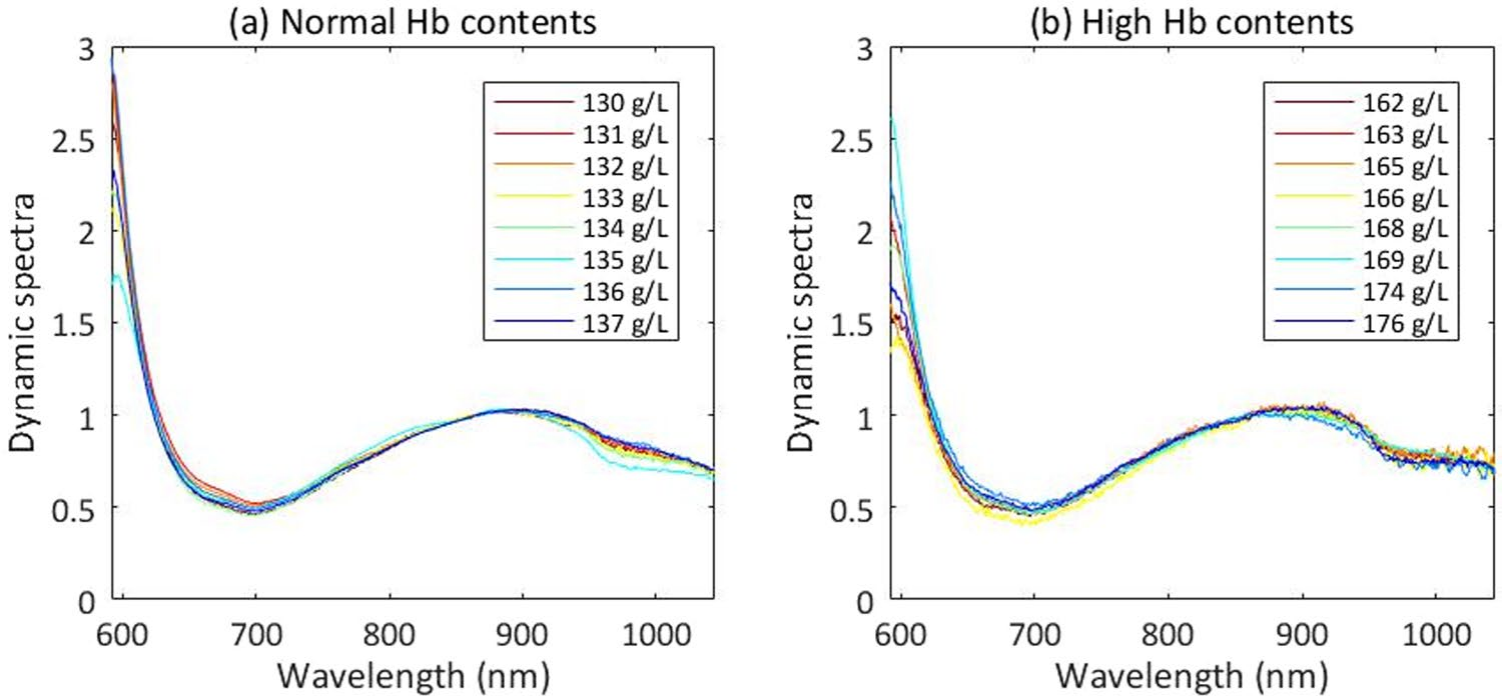
Dynamic spectra from randomly selected samples with different Hb contents. (**a**) Normal Hb contents. (**b**) High Hb contents.

If we observe the results carefully, we can see that grouping model 1 is better than grouping model 2, whether from the calibration or the prediction. We try to explain and find that dynamic spectra are not so smooth and have many burrs when Hb contents are high, as shown in Fig. 11. We can see that, though grouping modeling improves the prediction accuracy of high content range of Hb not so remarkably, it makes the overall prediction accuracy improved greatly.

In this paper, RSD of the non-invasive detection of Hb content is all smaller than 8%, though it can’t meet the standard for clinical application. Its main reason is that the non-invasive detection is interfered with human tissue (such as skin, muscle, fat)^38^. The new proposed method has pushed the accuracy closer to the gold standard, which demonstrated the effectiveness of grouping modeling sufficiently.

## Conclusions

Lambert-Beer’s law is the basis of quantitative analysis with absorption spectra and one important condition for its establishment is that the absorbing medium doesn’t scatter light. In non-invasive spectral detection of blood components, the strong scattering properties of blood result in the non-linear relationship between Hb content and dynamic spectrum. Therefore, a new method was proposed to decrease the influence of light scattering on the prediction accuracy of Hb: grouping modeling according to the content of Hb. Experimental results showed that the total RMSEP of two groups is smaller than that of the non-grouping modeling by 9.96% and RSDP smaller by 4.73% respectively. So, grouping modeling performs better in prediction accuracy of Hb than non-grouping modeling. This demonstrated that grouping modeling according to Hb content could correct non-linearity in a certain degree, thus improving the non-invasive prediction accuracy of Hb based on dynamic spectrum. This paper provides a new method and thinking of correcting the non-linearity caused by light scattering for the quantitative analysis with NIR absorption spectra.
